# Plant non-canonical peptides: From identification to mechanisms

**DOI:** 10.1016/j.xplc.2026.101739

**Published:** 2026-01-23

**Authors:** Shunxi Wang, Jinghua Zhang, Xiaojing Gao, Xiaodong Bao, Shanshan Liu, Ritian Qin, Benge Xin, Pengpeng Li, Bokai Zhang, Liuji Wu

**Affiliations:** 1 State Key Laboratory of High-Efficiency Production of Wheat–Maize Double Cropping, College of Agronomy, Henan Agricultural University, Zhengzhou, China; 2 Shanghai Applied Protein Technology Co., Ltd., Shanghai, China

**Keywords:** plant peptides, peptidogenomics, non-canonical peptides, receptor–ligand signaling, cross-disciplinary innovation

## Abstract

Plant peptides have emerged as key regulators of plant growth, development, immunity, and environmental adaptation. Early studies in crops demonstrate the potential application of certain peptides, such as systemin and plant elicitor peptides, to enhance disease resistance. Based on their structure and function, plant peptides are typically classified into canonical peptides, non-canonical peptides, and non-ribosomal peptides. Advances in peptidogenomics and mass spectrometry enable genome-wide discovery of numerous endogenous peptides, including those translated from untranslated regions and non-coding RNAs, greatly expanding the known plant peptidome. This review provides a comprehensive overview of plant peptides, including their classification, biosynthesis, and functional mechanisms in regulating diverse biological processes. Importantly, it systematically summarizes the historical development and recent advances in strategies for plant peptide identification. Despite substantial progress, peptide discovery and functional annotation remain challenging. We therefore propose that integrating high-throughput technologies, functional genomics, and synthetic biology will be essential for unlocking the full potential of plant peptides in crop improvement and cross-disciplinary innovation.

## Teaser

Plant peptides are vital signaling molecules that regulate plant growth, development, and environmental adaptation. This review provides a comprehensive overview of their classification, discovery approaches such as peptidogenomics, their key functional mechanisms, and emerging applications in agriculture. It further examines current challenges and future directions for harnessing plant peptides in crop improvement and biotechnology, offering insights to support cross-disciplinary innovation.

## Introduction

Plants, as sessile organisms anchored to their growth substrate, face numerous internal and external challenges throughout their life cycle. To cope with these stresses, they have evolved sophisticated defense systems that rely on intricate chemical signaling networks and physiological adaptations (Zhang et al., 2022; Leisner et al., 2023). The discovery of systemin—the first identified plant peptide—in tomato, and its role in triggering systemic acquired resistance against herbivores, underscores the importance of these bioactive molecules in plant immunity (Pearce et al., 1991). Subsequent studies have further revealed that peptides function as key regulators in diverse signaling pathways (Olsson et al., 2019; Chen et al., 2023, 2025; Lu and Xiao, 2024). For example, in *Arabidopsis thaliana*, CLAVATA3 (CLV3), a 12-amino-acid peptide that interacts with the CLV1/CLV2 receptor complex, regulates stem cell homeostasis in the shoot apical meristem by repressing WUSCHEL expression (Fletcher et al., 1999; Song et al., 2021). Similarly, phytosulfokines (PSKs) promote cell division through binding to PSK receptors in *Arabidopsis* (Kaufmann et al., 2021). To date, numerous peptides have been identified that contribute to enhanced crop resilience and productivity (Chen et al., 2023; Ali et al., 2024; Lu and Xiao, 2024; Sami et al., 2024; Xiao et al., 2025).

Plant peptides are broadly categorized into genome-encoded and non-genome-encoded peptides. Genome-encoded peptides include canonical peptides (CPs) and non-canonical peptides (NCPs), whereas non-genome-encoded peptides primarily comprise non-ribosomal peptides (NRPs). CPs are processed from larger precursor proteins, whereas NCPs originate from non-canonical open reading frames (ORFs) located within regions previously annotated as non-coding. In contrast, NRPs are synthesized by non-ribosomal peptide synthetases (NRPSs) and are independent of ribosomal translation and RNA metabolism (Tavormina et al., 2015; Süssmuth and Mainz, 2017; Wang et al., 2020; Chen et al., 2023). Advances in high-throughput sequencing and mass spectrometry (MS) have enabled comprehensive genome annotation and accelerated the discovery of peptide-encoding genes. For instance, thousands of NCPs have recently been identified in maize, among which 25 exhibit broad-spectrum antifungal activity (Wang et al., 2020; Tian et al., 2021).

Researchers have increasingly focused on the identification and functional characterization of plant peptides. This review provides a comprehensive overview of the major classes and characteristics of plant peptides, with particular emphasis on current identification strategies and their respective advantages and limitations. We further examine the mechanisms underlying the actions of key peptide classes. In addition, we discuss the major challenges facing plant peptide research and propose strategic directions for developing innovative approaches to enhance plant adaptability. Collectively, this review lays a foundation for decoding the roles of plant peptides and identifying emerging research opportunities. Such insights will support the development of strategies to enhance crop resilience, improve agronomic traits, and expand peptide-based applications in agriculture, biotechnology, and biomedicine.

## Classification of plant peptides

**Figure 1:**
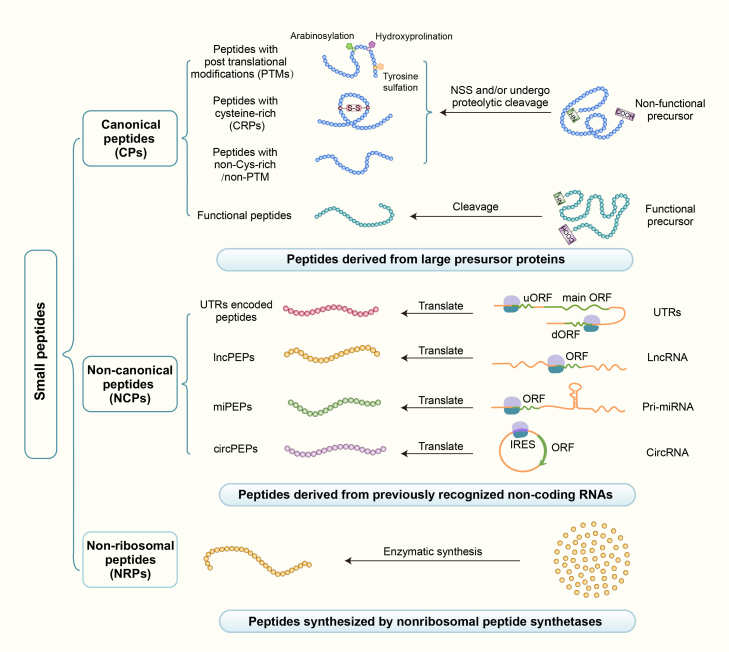
Classification and diversity of plant peptides. Plant peptides are broadly classified into three categories based on their origin: canonical peptides (CPs), non-canonical peptides (NCPs), and non-ribosomal peptides (NRPs). CPs are derived from annotated precursor proteins, including both functional and non-functional precursors. They can be further subdivided into post-translationally modified (PTM) peptides, cysteine-rich peptides, and non-cysteine-rich/non-PTM peptides. NCPs originate from genomic regions previously annotated as non-coding, including untranslated regions (UTRs), long non-coding RNAs (lncRNAs; yielding lncPEPs), primary microRNAs (pri-miRNAs; yielding miPEPs), and circular RNAs (circRNAs; yielding circPEPs). NRPs are biosynthesized independently of the ribosome via non-ribosomal peptide synthetases (NRPSs). NSS, N-terminal signal sequence.

Plant peptides are typically classified according to their origin, sequence features, structural characteristics, and biological functions. Broadly, they are grouped into three major categories: CPs, NCPs, and NRPs (Figure 1). This classification reflects both their translational origins and the molecular mechanisms underlying their biogenesis.

### Canonical peptides (CPs)

CPs are encoded by well-annotated protein-coding genes and are proteolytically processed from larger precursor proteins that typically contain a conserved N-terminal signal peptide, facilitating CP maturation and secretion (Olsson et al., 2019). Based on their origin and function, CPs can be subdivided into non-functional precursor-derived peptides and functional precursor-derived peptides.

Non-functional precursor-derived peptides are further categorized into three subtypes: post-translationally modified (PTM) peptides, cysteine-rich peptides (CRPs), and peptides lacking both cysteine residues and PTMs. PTM peptides acquire bioactivity through modifications such as proline hydroxylation, arabinosylation, or tyrosine sulfation; representative examples include Plant Peptides Containing Tyrosine Sulfation (PSYs) and Casparian Strip Integrity Factors (CIFs) (Matsubayashi, 2014; Takahashi et al., 2019; Kaufmann et al., 2021). CRPs—including RAPID ALKALINIZATION FACTORs (RALFs) and SMALL PHYTOCYTOKINES REGULATING DEFENSE AND WATER LOSS (SCREWs)—contain intramolecular disulfide bonds that are essential for maintaining structural stability and biological activity (Stegmann et al., 2017; Liu et al., 2022). Another well-characterized CRP, LATE ANTHER TOMATO 52 (LAT52), interacts with POLLEN-SPECIFIC RECEPTOR KINASE 2 (LePRK2) and plays a crucial role in pollen hydration and germination (Tang et al., 2002). Peptides that lack both cysteine residues and PTMs, such as systemin and plant elicitor peptides, nevertheless play central roles in plants (Pearce et al., 1991; Huffaker et al., 2006).

The functional precursor-derived peptides originate from precursor proteins that possess independent biological activity (Chen et al., 2014; Tavormina et al., 2015). A representative example is CAP-derived peptide 1 (CAPE1), which is processed from pathogenesis-related protein 1b (PR1b). CAPE1 exhibits distinct antibacterial and anti-herbivory activities (Chen et al., 2014) and plays a crucial role in salt-stress responses (Chien et al., 2015). Similarly, the soybean subtilase peptide (Gm-SUBPEP), a 12-amino-acid peptide derived from Glyma18g48580, functions as an endogenous elicitor that triggers innate immune responses and activates defense signaling pathways (Pearce et al., 2010; Yamaguchi and Huffaker, 2011). Collectively these earlier studies demonstrate that peptides derived from functional precursor proteins participate in diverse regulatory networks, thereby expanding the functional repertoire of plant defense and stress adaptation mechanisms.

### Non-canonical peptides (NCPs)

NCPs originate from small ORFs (sORFs) embedded within genomic regions previously annotated as non-coding, including untranslated regions (UTRs), long non-coding RNAs (lncRNAs), primary microRNAs (pri-miRNAs), and circular RNAs (circRNAs) (Chong et al., 2020; Wang et al., 2020). The first identified NCP, ENOD40, is a 10-amino-acid peptide encoded by an lncRNA (van de Sande et al., 1996). Advances in MS and ribosome profiling (Ribo-seq) have facilitated the identification of numerous NCPs across diverse plant species, revealing their widespread roles in regulating plant growth, development, and stress responses (Tian et al., 2021; Chen et al., 2023; Xiao et al., 2025).

Based on their origin, NCPs can be categorized into several subtypes, including UTR-derived peptides, lncRNA-encoded peptides, pri-miRNA-encoded peptides (miPEPs), and circRNA-encoded peptides, each with distinct biological functions. UTR-derived peptides are translated from sORFs located in the 5′ or 3′ UTRs of mRNAs. Genome-wide analyses across multiple species reveal that upstream open reading frames (uORFs) in 5′ UTRs are pervasive, and ribosome footprint profiling provides strong evidence that many of these uORFs are actively translated (Guo et al., 2023; Wu et al., 2024). Consequently, uORFs often function as *cis*-regulatory elements that repress translation of the main open reading frame (mORF) (Xu et al., 2017; Wang et al., 2024), although some enhance mORF translation efficiency under specific conditions (Guo et al., 2023). Similarly, downstream open reading frames (dORFs) can either enhance or repress mORF translation (Wu et al., 2020, 2024; Guo et al., 2023). Beyond their regulatory roles in modulating mORF translation, peptides derived from UTRs have been shown to perform independent biological functions in humans (Huang et al., 2021; Jayaram et al., 2021). However, their precise functions in plants remain elusive.

lncRNA-encoded peptides represent another major class of NCPs. Previously, lncRNAs were considered transcriptional noise due to the absence of canonical ORFs; however, accumulating evidence indicates that numerous sORFs within lncRNAs are translated into functional peptides (Zhu et al., 2023; Yi et al., 2024). POLARIS (PLS), which is involved in root and leaf vascular development, and KISS OF DEATH (KOD), which triggers programmed cell death in *Arabidopsis*, are well-characterized examples of lncRNA-encoded peptides (Casson et al., 2002; Blanvillain et al., 2011). In non-seed plants such as *Physcomitrella patens*, dozens of these peptides contribute to cell-fate specification and developmental phase transitions (Fesenko et al., 2019). Furthermore, genome-wide analyses have revealed that numerous plant lncRNAs harbor translatable sORFs, underscoring their widespread coding potential (Hanada et al., 2007; Lin et al., 2020).

miPEPs constitute another class of NCPs translated from sORFs embedded within pri-miRNA transcripts. pri-miRNAs are transcribed by RNA polymerase II and undergo sequential processing to generate mature miRNAs. In addition to their canonical role, pri-miRNAs often contain sORFs upstream of the stem–loop structure that encode functional miPEPs (Yadav et al., 2021). Notably, these peptides can enhance the transcription of their corresponding miRNA gene, reinforcing miRNA-mediated regulatory circuits and ultimately influencing diverse plant processes. For example, miPEP171b modulates lateral root development in *Medicago truncatula*, whereas miPEP858a regulates secondary metabolism in *Arabidopsis* (Lauressergues et al., 2015; Sharma et al., 2020).

circRNAs are a class of non-coding RNAs generated through noncanonical back-splicing events. Recent studies demonstrate that circRNAs harbor sORFs capable of encoding functional peptides in both mammals and plants (Yi et al., 2024; Pan et al., 2025). Although circRNA-encoded peptides appear to be relatively rare in plants, emerging evidence suggests that they represent an underappreciated class of regulatory molecules. For instance, the rice peptide WRKY9-88aa, encoded by a circRNA, enhances broad-spectrum disease resistance by promoting reactive oxygen species (ROS) production (Pan et al., 2025). Moreover, computational analyses predict that maize circRNAs possess peptide-coding potential, implying that circRNA-derived peptides may be more prevalent than currently recognized (Han et al., 2020).

### Non-ribosomal peptides (NRPs)

NRPs are synthesized by NRPSs in a ribosome-independent manner, thereby bypassing conventional mRNA translation. NRPSs assemble NRPs through a multistep process enabled by a modular architecture that permits the incorporation of both proteinogenic and non-proteinogenic amino acids, as well as diverse chemical modifications. As a result, NRPs exhibit substantial functional diversity and encompass a broad spectrum of biological activities, including antimicrobial, cytotoxic, and signaling functions (Greule et al., 2019).

Previously, NRPs have been extensively studied in microorganisms such as bacteria and fungi. However, accumulating evidence suggests that plants also produce a limited yet functionally important set of NRPs. Glutathione and phytochelatins are two well-characterized examples in plants. Glutathione, a tripeptide composed of glutamate, cysteine, and glycine, plays a central role in redox regulation, xenobiotic detoxification, and tolerance to oxidative stress (Noctor et al., 2012). Phytochelatins, synthesized from glutathione by phytochelatin synthase, are essential for heavy-metal chelation and homeostasis, particularly under cadmium, lead, or arsenic stress (Rauser, 1995; Cobbett, 2000).

## Identification of plant peptides

Historically, plant peptides were primarily identified through three approaches: bioassay-guided purification (Pearce et al., 1991; Huffaker et al., 2006), phenotype-based genetic analysis (Fletcher et al., 1999), and bioinformatic prediction based on sequence homology or signal peptides (Sawa et al., 2008; Huffaker et al., 2011). Although these strategies facilitated the discovery of early plant peptides, they yielded relatively few candidates and were largely restricted to well-characterized model systems such as *Arabidopsis*. Moreover, the inherently low abundance of peptides in plant tissues, together with their susceptibility to enzymatic degradation, presents significant challenges for extraction, purification, and downstream analyses.

Recent advances in high-throughput sequencing and MS technologies have enabled comprehensive, omics-scale profiling of endogenous peptides. In particular, the emergence of integrated approaches such as peptidogenomics has greatly accelerated the large-scale identification of small peptides, especially those encoded by non-canonical ORFs (Wang et al., 2020). This section therefore summarizes the historical progression and recent methodological advances in the identification of plant peptides, with an emphasis on their respective advantages and limitations.

### Bioassay-guided purification: A functional activity-driven method for isolating bioactive peptides from plant extracts

**Figure 2:**
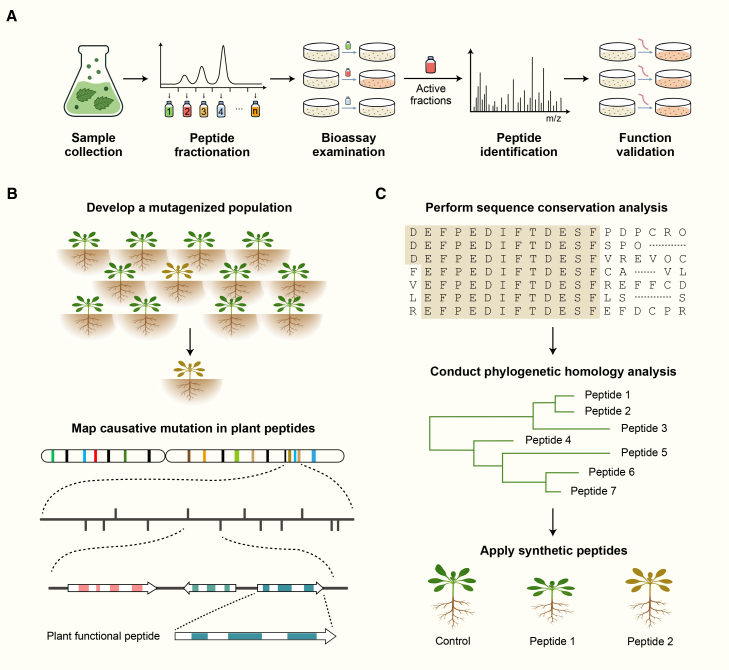
Traditional approaches for identifying plant peptides. **(A)** Workflow of peptide discovery using bioassay-guided purification. Peptide fractions are isolated and purified from plant extracts, and their sequences are analyzed using Edman degradation or liquid chromatography coupled with tandem mass spectrometry (LC–MS/MS). Synthetic peptides are subsequently used for functional validation. **(B)** Workflow of peptide discovery using phenotype-based genetic analysis. Positional cloning is performed to identify putative plant peptides. **(C)** Workflow of peptide discovery using a bioinformatics-based screening approach. Conserved sequences from known peptides are analyzed to predict novel candidates, whose functions are subsequently validated using synthetic peptides.

In 1991, the first plant peptide, systemin, was identified (Pearce et al., 1991), marking a milestone that enabled the subsequent isolation and functional characterization of additional plant peptides. Bioassay-guided purification remained the predominant strategy for peptide discovery for several decades. In this approach, peptides extracted from biological material are sequentially fractionated and then analyzed using techniques such as Edman degradation and MS to determine their amino acid sequences and confirm peptide identity (Skripnikov, 2023) (Figure 2A). To streamline the workflow and reduce the number of purification steps—including tissue disruption and fraction extraction—specialized cultures of submerged whole plants and isolated roots overexpressing the peptide precursors have been employed (Ohyama et al., 2008; Meng et al., 2012). In addition, to overcome the challenges associated with isolating low-abundance peptides, complex preparative protocols incorporating reverse-phase (C18) high-performance liquid chromatography were developed. The enriched fractions and synthetic peptides were subsequently administered to plant cell cultures or applied exogenously to seedlings to evaluate their biological functions (Skripnikov, 2023). Collectively, bioassay-guided strategies have been instrumental in validating peptide bioactivity. This integrated approach, which combines biochemical purification with functional screening, has led to the discovery of numerous regulatory peptides and continues to serve as a cornerstone of plant peptide research.

Many early-discovered peptides were isolated using this approach. For example, tobacco hydroxyproline-rich systemin (HypSys) I and II, which elicit wounding responses, and *Arabidopsis thaliana* plant peptide elicitor 1 (AtPep1), which triggers systemic antipathogen responses, were identified by screening for peptides capable of inducing extracellular alkalinization. HypSys I/II and AtPep1 were subsequently sequenced and characterized using Edman degradation and matrix-assisted laser desorption ionization (MALDI)–MS (Pearce et al., 2001b; Huffaker et al., 2006). Growth-modulating peptides such as RALF (Pearce et al., 2001a) and PSY1 (Amano et al., 2007) were also discovered using this traditional strategy. Together, these findings highlight the importance of bioassay-guided purification in identifying peptides with critical biological functions. Nevertheless, the inherently low abundance and small molecular size of peptides continue to present substantial challenges for their efficient separation and purification.

### Phenotype-based genetic analysis: A phenotype-driven approach for identifying functional plant peptides

Forward genetics is widely used to identify genes underlying specific phenotypes in an organism. This approach begins with phenotypic characterization, followed by mapping to identify the causative loci or genes. It has proven effective for discovering functional plant peptides (Figure 2B), including CLV3 (Fletcher et al., 1999). Similarly, the INFLORESCENCE DEFICIENT IN ABSCISSION (IDA)-encoding gene was identified through forward genetics and mapping in 2003 by analyzing a mutant in which floral organs remained attached after seed shedding. The gene was subsequently shown to encode a peptide essential for floral abscission (Butenko et al., 2003). More recently, genetic analysis identified a papain-like cysteine protease (TaRD21A) responsible for yellow mosaic virus resistance in wheat. Further investigation revealed that the TaRD21A-mediated antiviral response depends on the release of the Wip1 peptide (Liu et al., 2023). Together, these findings demonstrate that forward genetics can accelerate plant peptide discovery by enabling the identification of key genes and proteases involved in peptide production and function.

Forward genetics has also facilitated the discovery of NCPs encoded by sORFs in previously unannotated genomic regions. For example, a novel sORF located between two larger genes was identified in *Arabidopsis* from a short-root mutant exhibiting defective vascularization (Casson et al., 2002). Similarly, the *ROTUNDIFOLIA 4* (*ROT4*) gene, identified in 2004 from a leaf-shortening phenotype in *Arabidopsis*, was mapped to an intergenic locus (Narita et al., 2004). In addition, the discovery of the maize quantitative trait locus *qKDR1*, which silences the expression of the 31-amino-acid micropeptide microRPG1 (Yu et al., 2025), illustrates how forward genetics can uncover novel peptides through their upstream regulatory elements. Despite these advances, several challenges remain, including the genetic redundancy often exhibited by peptides and their diverse genomic origins. These limitations underscore the need for specialized biochemical and omics-driven methodologies to further advance plant peptide discovery.

### Bioinformatic analysis: A computational data-mining strategy for predicting putative peptides

Bioinformatics enables peptide prediction based on sequence features, including peptide-specific motifs and N-terminal signal peptides. Primary computational strategies for discovering plant peptides typically involve searching for sequences corresponding to known peptides or those sharing similarity with reference sequences (Figure 2C). In this context, tools such as BLAST are commonly used to align query sequences against databases of known plant peptides (Lease and Walker, 2010). Parameter optimization, such as reducing e-value thresholds, can enhance sensitivity and improve the detection of short or divergent sequences. For example, conserved motifs within precursor proteins—such as those of CLAVATA3/EMBRYO SURROUNDING REGION-RELATED (CLE) peptides—have facilitated the identification of homologs and orthologs across diverse plant species (Kucukoglu and Nilsson, 2015). Similarly, homologs of the RALF peptide family have been identified in several crops, including potato, wheat, barley, and soybean (Campbell and Turner, 2017). Although this strategy has been effective in identifying key CP members, its utility for discovering novel peptides, particularly NCPs, remains limited.

### Peptidomics: An MS-based approach for discovering plant peptides

Peptidomics is an MS-based high-throughput approach used to identify endogenous peptides without proteolytic digestion. First applied in insects in 2001 (Clynen et al., 2001; Verhaert et al., 2001), it was subsequently adapted for plants, beginning with *Arabidopsis* in 2008 (Ohyama et al., 2008). Unlike classical bottom-up proteomics, peptidomics captures peptides in their native, biologically active forms while preserving their N and C termini and PTMs, making it particularly suitable for investigating peptide processing and signaling *in vivo*.

Peptidomics has facilitated the discovery of plant peptides involved in stress responses, immunity, and developmental processes. For instance, comparative peptidomic profiling in tomato identified wound-induced peptides (Chen et al., 2014), whereas high-throughput peptidomics in *Arabidopsis* revealed immune-responsive fragments regulated by thimet oligopeptidases (Iannetta et al., 2022). Moreover, incorporating machine learning into peptidomic pipelines has improved peptide identification and activity prediction (Perpetuo et al., 2021). Recently, deep peptidomic analysis across 13 maize tissues identified 6100 non-redundant endogenous peptides (Ali et al., 2024), substantially expanding the known plant peptidome.

Beyond the classical workflow that bypasses proteolytic digestion, enzyme-assisted strategies have been employed to identify structurally unique or cyclic peptides in plants (Hellinger et al., 2015). This flexibility broadens the applicability of peptidomics for capturing peptide classes with distinct physicochemical properties. Despite these advantages, peptidomics lacks the genomic context required for *de novo* peptide discovery, particularly for NCPs derived from unannotated or intergenic regions.

### Peptidogenomics: An integrated strategy for enhanced plant peptide identification

Conventional peptidomics has substantially advanced plant peptide discovery but remains limited in its ability to uncover NCPs. To address this gap, an integrated strategy combining MS with genome-derived databases was developed to enable genome-scale identification of plant peptides (Wang et al., 2020). Using this approach, we identified 1993 and 1860 NCPs in maize and *Arabidopsis*, respectively, along with numerous peptides corresponding to annotated coding regions. These NCPs originated from diverse genomic loci, suggesting active translation of previously unannotated regions. Notably, many were enriched within genomic intervals associated with complex agronomic traits and domestication, supporting their functional relevance in regulatory networks and adaptive evolution.

**Figure 3:**
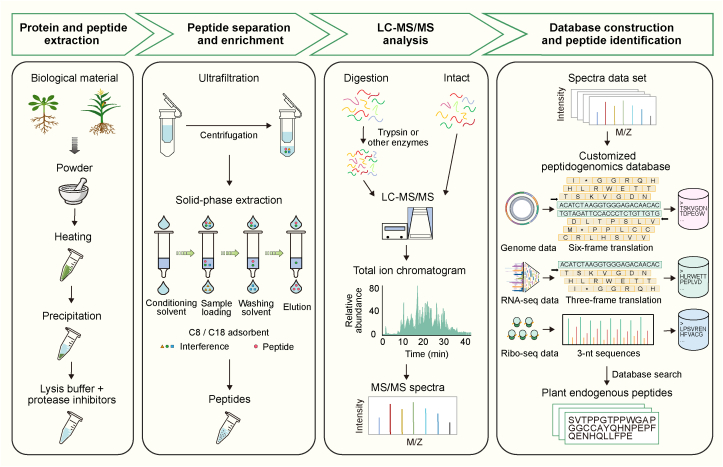
Generalized peptidogenomic workflow for identifying endogenous plant peptides. The peptidogenomics pipeline integrates MS-based peptidomics with genome- and transcriptome-derived databases to enable comprehensive and accurate identification of endogenous plant peptides. Peptides are first extracted from plant tissues and enriched using techniques such as ultrafiltration and solid-phase extraction (SPE). They are then analyzed directly or following enzymatic digestion using liquid chromatography coupled with tandem MS (LC–MS/MS). For peptide identification, customized sequence databases are generated through six-frame translation of the genome. Additionally, databases derived from three-frame translation of transcriptome assemblies or Ribo-seq–defined translation regions are employed to further facilitate peptide identification.

Peptidogenomics has since been applied across multiple plant systems. The approach has enabled the identification of antimicrobial peptides in *Amaranthus tricolor* (Moyer et al., 2021), genome-scale endogenous peptides in grape (Pei et al., 2022), and phased secondary small interfering RNA (phasiRNA)-encoded peptides in maize (Han et al., 2024). Collectively, these studies underscore the versatility and power of peptidogenomics in expanding the known plant peptidome. A typical peptidogenomic workflow comprises four core steps (Figure 3): (1) sample pretreatment and peptide extraction, (2) peptide separation and enrichment, (3) liquid chromatography coupled with tandem mass spectrometry (LC–MS/MS), and (4) customized database construction followed by peptide identification.

(1) **Sample pretreatment and peptide extraction**. Efficient extraction of endogenous peptides is essential for minimizing protease-mediated degradation and preserving native structure. Various strategies are used to inactivate proteases and stabilize peptides, including physical methods such as rapid heating and chemical treatments such as acidified lysis buffers (pH < 3) and protease inhibitors (Chen et al., 2014; Wang et al., 2020). In addition, organic solvent-based precipitation methods (*e.g.*, trichloroacetic acid–acetone, methanol/chloroform) help remove high-molecular-weight proteins while retaining soluble peptides (Dufresne et al., 2018; Wang et al., 2020). In peptidogenomic workflows, combining rapid heating with protease inhibitors effectively preserves peptides. Beyond acidified lysis buffers, urea-based lysis buffers are also employed to denature cellular proteins and inactivate proteases during the extraction of small peptides (Pei et al., 2022).
(2) **Peptide separation and enrichment**. Following extraction, enrichment is performed to isolate low-abundance peptides from complex plant matrices prior to MS analysis. Ultrafiltration with membranes of defined molecular weight cutoffs is commonly used to enrich small peptides based on size and physicochemical properties. Solid-phase extraction (SPE) is subsequently applied to further purify peptide mixtures and eliminate matrix-derived impurities. In our workflow, ultrafiltration was combined with SPE to efficiently remove contaminants while retaining endogenous peptides. This integrated enrichment strategy enhances detection sensitivity and facilitates the identification of numerous plant peptides.
(3) **LC–MS/MS analysis**. LC–MS/MS plays a central role in peptidogenomics by enabling the direct identification of endogenous peptides. Because plant peptides are typically short and have low molecular weights, top-down MS is particularly suitable, as it analyzes intact peptides without enzymatic digestion. This approach avoids ambiguities caused by shared or missing fragments, provides higher sequence coverage, and generates more accurate structural information. Bottom-up MS, which involves enzymatic digestion prior to analysis, can also be used to identify plant peptides (Moyer et al., 2021). However, digestion of short peptides generates limited fragment ions, leading to fewer peptide-spectrum matches and reduced sequence coverage. Consequently, bottom-up MS is generally less effective for small endogenous peptides but can serve as a complementary strategy when integrated with a top-down workflow to enhance peptide identification.
(4) **Customized database construction and peptide identification**. In MS-based peptidogenomics, acquired spectra are matched against theoretical spectra in reference databases to identify endogenous peptides. To ensure high-confidence assignments, spectral matching is supported by two additional layers of evidence: retention time alignment, which helps eliminate mismatched identifications, and high-quality fragment ion analysis, which provides definitive sequence validation. Because traditional protein databases are largely derived from annotated coding sequences, NCPs are often overlooked, underscoring the need for more comprehensive and customized databases. Our peptidogenomic strategy addresses this limitation by generating sequence databases through six-frame translation of the entire genome. This approach enables unbiased, genome-scale peptide identification, including peptides derived from intergenic, intronic, and UTR regions. Consequently, peptidogenomics has uncovered peptides originating from previously unannotated regions and with potential novel functions, thereby expanding the known plant peptidome.

Alternative databases further support this strategy. For example, three-frame translation of transcriptome assemblies from RNA-sequencing data has been used to identify peptides (Moyer et al., 2021). Similarly, translated ORFs identified through Ribo-seq can be incorporated to generate more accurate custom databases for peptide identification (Liang et al., 2021). *In silico* translation of targeted sequences can also facilitate the construction of specialized databases, such as those for phasiRNA-encoded peptides (Han et al., 2024). Continued development of refined, context-specific peptide databases will further enhance the sensitivity and resolution of peptidogenomic analyses. Despite these advances, no standardized framework currently exists for peptide annotation, particularly for NCPs, and existing methods retain notable limitations. Integrating multi-omics datasets—including transcriptomics, translatomics, proteomics, and genomics—may help overcome these challenges and provide a more comprehensive understanding of plant peptides.

In conclusion, research on plant peptide identification reflects methodological advances driven by progress in analytical technologies. The field began with the discovery of systemin, followed by the widespread use of bioassay-guided purification, which enabled the identification of several plant peptides. However, this approach remains challenging due to the low abundance and small size of peptides in plant tissues. Subsequently, phenotype-based genetic analysis, grounded in classical genetics and mutant screening, was employed to uncover functional peptides. Nevertheless, functional redundancy has limited the number of peptides identified through this strategy. With continued progress in sequencing technologies and bioinformatics, numerous peptides have been discovered across diverse plant species. A key limitation of bioinformatic analysis is its reliance on consensus motifs, which facilitates the identification of additional members within known peptide families but restricts the discovery of NCPs that often exhibit low sequence conservation. Advances in MS have further enabled large-scale peptide identification through peptidomics; however, this technique remains less effective for detecting NCPs. More recently, the integrated strategy of peptidogenomics has emerged as a powerful approach for enhancing the identification of both CPs and NCPs in plants. Notably, combining peptidogenomics with translatome data holds considerable promise for accelerating the discovery of novel plant peptides.

## Mechanisms underlying plant peptide functions

Understanding how small peptides exert their biological functions is essential for harnessing their potential in crop improvement and stress resilience. Plant peptides operate across multiple regulatory layers, from activating cell-surface receptors to modulating post-transcriptional and translational processes, underscoring their versatility as signaling mediators. In this section, we discuss the principal mechanisms by which plant peptides influence cellular functions, including receptor interactions, miRNA modulation, translational control, and protein-level interactions.

### Extracellular mechanisms: Secreted peptides act as ligands for receptor-like kinases

**Figure 4:**
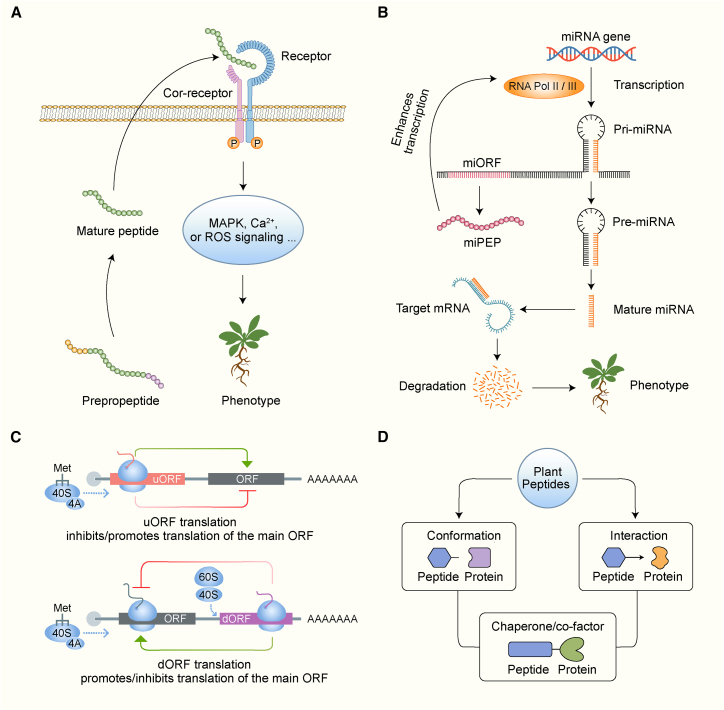
Representative molecular mechanisms underlying peptide functions in plants. **(A)** Mature peptides derived from prepropeptides are secreted into the apoplast, where they bind receptor-like kinase (RLK) complexes and initiate intracellular signaling pathways such as mitogen-activated protein kinase (MAPK) activation, Ca^2+^ influx, and reactive oxygen species (ROS) bursts, ultimately regulating plant growth, immunity, and environmental responses. **(B)** MicroRNA-encoded peptides (miPEPs) positively regulate the transcription of their corresponding pri-miRNA genes, thereby enhancing mature miRNA accumulation and promoting target gene silencing. **(C)** Peptides encoded by upstream and downstream open reading frames (uORFs and dORFs) modulate the translation of main ORFs (mORFs). uORF-derived peptides repress translation via ribosomal stalling, whereas dORF-derived peptides enhance translation by promoting ribosome reinitiation or mRNA looping. **(D)** Peptides function through direct peptide–protein interactions, serving as structural regulators or molecular chaperones that influence protein localization, stability, or activity.

Many plant peptides function as extracellular signals by binding to plasma membrane-localized receptors and activating downstream signaling cascades that regulate development, defense, and environmental responses (Figure 4A). Most receptors mediating peptide signaling belong to the leucine-rich repeat receptor-like kinase (LRR-RLK) family. These transmembrane proteins are characterized by an extracellular leucine-rich repeat domain, a single transmembrane helix, and a cytoplasmic kinase domain (Furumizu and Aalen, 2023). Other receptor-like kinases (RLKs), including lectin RLKs, lysine-motif RLKs, and S-domain RLKs, have also been implicated in peptide recognition (Sanabria et al., 2008; Ngou et al., 2024). Upon ligand binding, these receptor complexes transduce extracellular cues into intracellular signals that ultimately regulate transcriptional programs and gene expression. A well-characterized peptide–receptor module involves ROOT MERISTEM GROWTH FACTORs (RGFs), which interact with RGF1 INSENSITIVE (RGI) receptors to control root development and immune responses in *Arabidopsis* (Ou et al., 2016; Wang et al., 2021). Similarly, PAMP-INDUCED SECRETED PEPTIDEs (PIPs) bind to RECEPTOR-LIKE KINASE 7 (RLK7), thereby activating immune responses and enhancing salt-stress tolerance (Hou et al., 2014; Zhou et al., 2022). Recent studies have also identified multiple peptide–receptor pairs in crop species. For example, REGENERATION FACTOR 1 (REF1) interacts with PEPR1/2 ORTHOLOG RECEPTOR-LIKE KINASE 1 (PORK1) to regulate local defense and regenerative responses in tomato (Yang et al., 2024). In wheat, the Delta-like PCK (DEP) peptide and its receptor DEP RECEPTOR 1 (DEPR1), as well as the DUAL CYSTEINES IN C TERMINUS 1 (DCC1) peptide and its receptor DCC RECEPTOR 1 (DCCR1), play important roles in immunity and disease resistance (Wang et al., 2025; Yin et al., 2025).

Both the primary amino acid sequence and PTMs regulate the specificity of peptide–receptor interactions (Matsubayashi, 2014; Kaufmann et al., 2021). Structural features such as disulfide bonds, crucial for peptides such as RALFs and SCREWs, stabilize peptide–receptor conformations and regulate immune signaling (Du et al., 2016; Liu et al., 2022). Typically, ligand binding induces RLKs to dimerize with a co-receptor, triggering autophosphorylation and activation of downstream kinases such as mitogen-activated protein kinases (MAPKs) and calcium-dependent protein kinases (CDPKs); this process forms a critical link between extracellular cues and intracellular transcriptional or hormonal responses (Furumizu and Aalen, 2023). SOMATIC EMBRYOGENESIS RECEPTOR KINASEs (SERKs), such as BRASSINOSTEROID INSENSITIVE 1 (BRI1)-ASSOCIATED RECEPTOR KINASE 1 (BAK1) (Lalun and Butenko, 2025), along with other common co-receptors such as SUPPRESSOR OF BIR1 (SOBIR1), are among the most frequently involved co-receptors in peptide perception (Albert et al., 2015). Activated receptor kinases subsequently modulate diverse cellular processes, including calcium ion (Ca^2+^) fluxes and ROS generation (Hohmann et al., 2017).

### Nuclear mechanisms: Transcriptional regulation via miPEPs

Certain small peptides, known as miPEPs, function within the nucleus to regulate transcription of their corresponding pri-miRNA (Figure 4B). For example, miPEP165a in *Arabidopsis* and miPEP171b in *Medicago truncatula* upregulate the transcription of their respective pri-miRNAs, thereby promoting the silencing of downstream target genes (Lauressergues et al., 2015, 2022). This feedback loop reinforces miRNA-mediated regulatory pathways in a sequence- and position-dependent manner. Notably, transcriptional inhibitors such as cordycepin abolish this effect, indicating that miPEPs enhance *de novo* transcription of pri-miRNAs rather than RNA stability. A recent study further demonstrated that miPEP858 directly interacts with the promoter of the pri-miRNA858 gene, with the C-terminal region of miPEP858 playing a crucial role in regulating the expression of both miR858 and its target gene (Gautam et al., 2025). Collectively, these findings reveal a direct mechanism through which peptides reinforce gene-silencing pathways in plants.

### Translational control: Regulation of protein synthesis via uORFs and dORFs

Plant peptides regulate gene expression at the translational level through uORFs and dORFs (Figure 4C). During translation initiation, the 43S pre-initiation complex is recruited to mRNA by the cap-associated eIF4F complex and scans from the 5′ end for an optimal initiation codon. Under specific conditions, the 80S ribosome preferentially assembles at the uORF start site, reducing ribosome access to the mORF and thereby lowering protein output from the mORF (Mou et al., 2025). For example, under low-energy conditions, translation of the uORF in bZIP11 induces ribosome stalling, resulting in the downregulation of this key transcription factor involved in stress adaptation (Rahmani et al., 2009; Zhang et al., 2020c). Similar mechanisms govern the translation of auxin response factors and contribute to ribosomal mutant phenotypes (Rosado et al., 2012). These peptides function by forming secondary structures or directly interacting with the ribosome, thereby inhibiting codon scanning or translation reinitiation (Lin et al., 2019). Although most uORFs suppress downstream translation, some enhance translation or function independently, highlighting diverse regulatory modes (Yang et al., 2020).

Conversely, dORFs located in the 3′ UTR can facilitate mORF translation by enhancing mRNA stability or promoting ribosome recycling (Wang et al., 2020; Guo et al., 2023). While dORF translation has been investigated in animal systems, its functional significance in plants remains largely unexplored. Nonetheless, the identification of dORFs challenges the classical monocistronic view of plant mRNAs and underscores additional layers of translational control.

### Intracellular mechanisms: Interaction with other proteins

Many plant peptides perform intracellular functions by directly interacting with proteins. These peptides localize to organelles such as the endoplasmic reticulum (ER) or Golgi apparatus, where they influence protein trafficking, complex assembly, and metabolic activity. For example, TWISTED SEED 1 (TWS1), localized to the ER in *Arabidopsis*, interacts with proteins involved in secretion and PTMs (Fiume et al., 2016). Similarly, AtZSP1 modulates organ size by interacting with the ER-localized UDP-GlcNAc transporter ROCK1, thereby affecting glycoprotein processing (Zeng et al., 2022).

Certain peptides also function as chaperones or co-factors, stabilizing enzymatic complexes. In pollen grains, the peptide RALF4 acts as a cell wall component that guides pollen-tube elongation through coordinated mechanical and signaling processes (Moussu et al., 2023). In legume–rhizobia symbiosis, nodule-specific cysteine-rich peptides bind rhizobial proteins, modulating bacterial differentiation and maintaining symbiotic compatibility (Pan and Wang, 2017). Additionally, a recent study showed that the microRPG1 peptide regulates kernel dehydration rate in maize by modulating the expression of ethylene-insensitive genes (Yu et al., 2025). Together, these findings demonstrate that plant peptides function as both regulators and effectors in protein–protein interactions (Figure 4D).

## Challenges and future perspectives

Over the past decade, research on plant peptides has advanced substantially. The identification of NCPs, progress in peptidogenomics, and growing interest in peptide-based applications have revealed a complex and still underexplored research landscape, highlighting the potential of these bioactives for crop improvement. To fully realize this potential, however, challenges associated with peptide identification must be addressed. This section outlines the major bottlenecks in peptide discovery and functional analysis and proposes directions for future research and translational innovation.

### Technical challenges in peptide discovery and profiling

Comprehensive identification of plant peptides, particularly NCPs, remains technically challenging. Compared with proteins, peptides are shorter, often less abundant, and highly sequence-diverse, rendering them more susceptible to degradation and difficult to capture from complex tissue extracts. Conventional LC–MS/MS platforms face additional limitations due to overlapping isoforms and PTMs that alter peptide mass and charge states. Moreover, the lack of plant-specific peptide databases represents a major bottleneck (Hellinger et al., 2023). Protein repositories, such as UniProt, provide partial support; however, genome-wide annotations of sORFs and validated peptide datasets remain scarce. These constraints significantly hinder the identification of novel peptides, particularly those encoded by intergenic, intronic, or *de novo* transcribed regions. Recent integrated approaches, such as peptidogenomics—which combines peptidomics with genomics, transcriptomics, and translatomics—are proving increasingly powerful (Chen et al., 2023). In parallel, machine-learning-based tools are expected to accelerate peptide discovery by improving the sensitivity and accuracy of detection strategies.

### Complexities in functional characterization

Elucidating the biological roles of plant peptides remains a major challenge in the field. The functions of most plant peptides, particularly NCPs, are still unknown. Functional redundancy and signaling overlap among peptide family members often obscure loss-of-function phenotypes, whereas ectopic overexpression often leads to pleiotropic or non-specific effects. Moreover, peptide activity frequently varies across developmental stages, environmental conditions, and tissue types. Peptides may also interact with multiple receptors, participate in ligand–receptor co-evolution, and indirectly modulate transcriptional or translational networks, complicating efforts to establish clear genotype–phenotype relationships (Xiao et al., 2025; Zhang et al., 2025). These complexities necessitate advanced genetic and biochemical strategies for functional characterization. For instance, CRISPR-based multiplex genome editing enables simultaneous targeting of redundant peptide-encoding genes, while inducible expression systems provide temporal control over peptide activity. In parallel, systematic mapping of peptide–receptor interactions and functional analyses will help clarify receptor specificity and downstream signaling pathways. Additionally, reliance on a single reference genome for peptide identification within a species may result in missed peptides absent from that genome and overlook natural sequence diversity. Establishing pan-genomes or super-pan-genomes is therefore expected to accelerate the identification of core plant peptides. Furthermore, investigating peptide variation and conducting comparative analyses across species within a genus—or across genera—will broaden our understanding of peptide diversity and facilitate the generation of novel peptide-encoding alleles for improving complex quantitative traits.

### Barriers to the agricultural application of exogenous peptides

Despite growing interest in synthetic peptides, their use in agriculture as biostimulants or plant protectants remains limited. One major challenge is the high cost associated with peptide synthesis or production, particularly for peptides requiring PTMs, such as tyrosine sulfation or hydroxyproline arabinosylation. Moreover, peptides are often unstable under field conditions due to protease activity, photolysis, and microbial degradation (Xiao et al., 2025). Efficient delivery of peptides to target tissues presents another significant hurdle. Foliar sprays often result in poor peptide penetration, whereas root applications encounter diffusion and adsorption barriers. In addition, regulatory frameworks for peptide-based products remain underdeveloped in many regions, creating uncertainty around commercialization and large-scale field deployment. Although exogenous peptides hold promise for enhancing plant resistance, their application may inadvertently affect plant growth and development (Zhang et al., 2020a). To overcome these barriers, future research should focus on developing scalable production platforms, such as plant cell factories or engineered microbes, and clarifying the critical links between growth and disease resistance to improve stress tolerance with minimal yield penalties. Advanced delivery systems—including encapsulation technologies and slow-release formulations—should also be explored. Furthermore, engineered peptides optimized for receptor binding through artificial intelligence–guided design may enhance both selectivity and potency. Collectively, progress across these areas will strengthen the discovery and deployment of peptides with agricultural potential. Addressing challenges associated with translational research will be essential for fully realizing the promise of plant peptides in sustainable crop improvement.

### Outlook and opportunities

(1) Despite existing challenges, the future of plant peptide research is highly promising. Advances in high-resolution MS, AI-guided structure-based functional prediction, and long-read transcriptomic sequencing are expected to facilitate the generation of comprehensive peptide atlases across species and environmental conditions. Emerging evidence indicates that many peptides originate *de novo* from non-coding regions and are subjected to strong natural selection, particularly those associated with environmental adaptation. The co-evolution of signaling peptides and their cognate receptors further enhances specificity, positioning these molecules as ideal candidates for synthetic biology applications.
(2) Plant-based cell factories, algae, and engineered microbes are being explored as scalable platforms for peptide production. To support effective field deployment, future efforts should prioritize innovative delivery strategies, including encapsulation, polymer-based slow-release systems, and foliar nanocarriers, to protect peptides from environmental degradation and enable targeted release.
(3) Beyond these advances, integrating multiscale and spatiotemporal frameworks will be essential for understanding peptide signaling. Most studies have focused on locally acting peptides; however, accumulating evidence suggests that peptides can function as mobile signals, coordinating long-distance responses through the phloem or xylem (Zhang et al., 2020b). Approaches such as spatial transcriptomics, single-cell omics, and live-cell imaging will be critical for resolving these dynamics and elucidating peptide activity across tissues and environmental contexts.
(4) Moreover, peptides are increasingly recognized as nodes linking diverse signaling pathways, including hormones (*e.g.*, auxin and abscisic acid), calcium fluxes, and ROS, thereby integrating developmental and stress signals (Xiao et al., 2025). Future research should prioritize mapping these intersections and constructing dynamic network models that capture the mechanisms underlying peptide-mediated control. Integrating computational modeling, structural biology, and functional peptidomics will help decipher peptide signaling networks. These efforts will further support the design of synthetic receptors, programmable peptide modules, and tailored regulatory circuits for precision agriculture.
(5) Plant peptides are also expected to have relevance in fields such as biomedicine, biosensing, and materials science. Accordingly, these bioactives offer opportunities for cross-disciplinary innovation and broad translational research impact.

## Funding

This work was supported by the 10.13039/501100001809National Natural Science Foundation of China (nos. U22A20474 and 32172073), the 10.13039/501100012166National Key Research and Development Program of China (no. 2022YFD1201802), and the Key Scientific and Technological Project of the Henan Province Department of Science and Technology, China (no. 242102111129).

## Acknowledgments

We thank Dr. Anguo Sun for helpful discussions. The authors declare no competing interests.

## Author contributions

L.W., S.W., J.Z., and X.G. designed the review outline. S.W., J.Z., X.G., X.B., R.Q., B.X., P.L., B.Z., S.L., and L.W. wrote the majority of the manuscript. S.W., J.Z., X.G., B.Z., X.B., S.L., and L.W. prepared the figures. L.W., S.W., and J.Z. revised the manuscript. All authors read and approved the final manuscript.
